# A multidimensional review of the cash management problem

**DOI:** 10.1186/s40854-023-00473-7

**Published:** 2023-03-15

**Authors:** Francisco Salas-Molina, Juan A. Rodríguez-Aguilar, Montserrat Guillen

**Affiliations:** 1 Universitat Politècnica de València, Ferrándiz y Carbonell, 03801 Alcoy, Spain; 2 IIIA-CSIC, Campus UAB, 08913 Cerdanyola, Spain; 3 Universitat de Barcelona, Av. Diagonal, 690, 08034 Barcelona, Spain

**Keywords:** Cash management, Models, Dimensions, Review

## Abstract

In this paper, we summarize and analyze the relevant research on the cash management problem appearing in the literature. First, we identify the main dimensions of the cash management problem. Next, we review the most relevant contributions in this field and present a multidimensional analysis of these contributions, according to the dimensions of the problem. From this analysis, several open research questions are highlighted.

## Introduction

Cash managers must make daily decisions about the number of transactions between cash holdings and any other type of available investment asset. On the one hand, a certain amount of cash must be kept for operational and precautionary purposes. On the other hand, idle cash balances may be invested in short-term assets such as interest-bearing accounts or treasury bills for profit. Since Baumol (1952), several cash management models have been proposed to address the cash management problem(CMP).

Keynes (1936) initially identified three motives for holding cash: the transaction motive, the precautionary motive and the speculative motive. Other authors have added other motives for holding cash, such as the agency motive (Jensen 1986) or tax motive (Foley et al. 2007). More recently, other authors have highlighted other determinants of corporate cash policies (e.g., Gao et al. (2013) and Pinkowitz et al. (2016)). As a result, the first objective of this study is to review the literature related to CMP from an economic and financial perspective, derived from the analysis of the main motives for holding cash.

While most cash management literature stems from the seminal paper by Baumol (1952), many cash management works approach CMP from a decision-making perspective. Our second objective is to review the literature related to CMP from a decision-making perspective, considering models proposed by different researchers to deal with cash when the ultimate goal is to elicit a cash management policy, namely, a temporal sequence of transactions between accounts.

To the best of our knowledge, only three surveys on cash management have been published since the 1950s. Gregory (1976) covered the beginning of the cash management literature including the important works by Baumol (1952) and Miller and Orr (1966). Ten years later, Srinivasan and Kim (1986) extended the analysis to models not considered by Gregory (1976). Finally, da Costa Moraes et al. (2015) reviewed several stochastic models since the 1980s. However, there is a lack of taxonomy for classifying models and identifying open research questions in cash management.

Within the context of CMP, from a decision-making perspective, we propose a taxonomy based on the main dimensions of the cash management problem: (i) the model deployed, (ii) the type of cash flow process considered, (iii) the particular cost functions used, (iv) the objectives pursued by cash managers, (v) the method used to set the model and solve the problem, and (vi) the number of accounts considered. These six dimensions provide a sound framework to classify the cash management models proposed in the literature. Here, we focus on the most relevant models in terms of number of citations. For a comprehensive review, we refer interested readers to Gregory (1976), da Costa Moraes et al. (2015) and Srinivasan and Kim (1986).

Our taxonomy helps researchers use a common framework to establish cash management areas. In addition, our multidimensional analysis enhances the understanding of the cash management problem, making it easier to identify open research questions. Note that the multidimensional framework described in this paper is not limited to the six dimensions mentioned above. Researchers may extend the number of dimensions, thereby enriching the analysis of the cash management problem.

The remainder of this paper is organized as follows. In "Motives for holding cash and related literature" in section, we consider the main motives for holding cash and review the literature related to CMP from an economic and financial perspective. In " A multidimensional taxonomy of the cash management problem" in section, we introduce and motivate the six dimensions of the CMP that define our taxonomy proposal. In "A review of the main contributions to the cash management problem" in section, we review the most relevant contributions to CMP from a decision-making perspective. Next, " A multidimensional analysis of the cash management problem" in section, we perform a comparative analysis of alternative cash management models that are directly linked to "Open research questions in cash management" in section, which identifies several open research questions in cash management. Finally, "Concluding remarks" in section concludes the paper.

## Motives for holding cash and related literature

In this section, we consider the main motives for holding cash and review the literature related to CMP from an economics and finance perspective. We first consider the three motives for holding cash, initially identified by Keynes (1936), as follows:

1. The transaction motive, which is the need for cash for the current transaction of personal and business exchanges.
2. The precautionary motive, which is the desire for security as the future cash equivalent of a certain proportion of total resources acts as a financial reserve.
3. The speculative motive or the object of securing profit from knowing better than the market what the future will bring forth. The goal is to take advantage of future investment opportunities.

Later, Jensen (1986) argued that managers tend to accumulate cash rather than increase payouts to shareholders because of agency motives. Cash holdings may act as a buffer to cover eventual bad management decisions. One possible reason for this behavior is information asymmetry. Information is distributed asymmetrically throughout the organization; thus, managers usually have an advantage over shareholders in handling specific events because of information asymmetry (Eisenhardt 1989; Dierkens 1991). In addition, managers have an incentive to make the company bigger when compensation is linked to the size of the company, even when the company has poor investment opportunities. The motive for holding cash stems from the financial implications of agency theory (Jensen and Meckling 1976; Fama 1980; Fama and Jensen 1983; Eisenhardt 1989). In this theory, the firm is viewed as a set of contracts among the factors of production, in which each one is motivated by self-interest (Fama 1980). Consequently, the relationship between corporate managers (including cash managers) and owners presents friction due to conflicts of interest. The concept of agency costs defined by Jensen and Meckling (1976) is derived from an agency relationship in which managers and owners present divergences that result in monitoring costs, bonding costs to avoid certain actions, and other residual losses. One of these divergences relates to cash holdings. For example, consider that cash outflows to shareholders in the form of dividends reduce resources under managers’ control.

Kaplan and Zingales (1997) investigated the relationship between sensitivity of investment to cash flow and financing constraints, expressed as the differential cost between internal and external finance. They found that even though investment is sensitive to cash flow for the vast majority of firms analyzed, investment-cash flow sensitivities do not increase monotonically with the degree of financing constraints. Most of the firms analyzed could increase their investment if they choose to do so, thus providing further evidence of the agency motive for holding cash. Contrary to what was thought before, the authors concluded that higher sensitivities cannot be interpreted as evidence that firms are more financially constrained.

Leland (1998) argued that the key insight by Jensen and Meckling (1976) is that the firm’s choice of risk may depend on capital structure, hence challenging the Modigliani and Miller (1958, 1963) assumption that investment decisions are independent of capital structure. Consequently, Leland (1998) proposed integrating both approaches to derive the optimal capital structure of a firm. The model reflects the interaction of different cash flow policies, namely, financing decisions and investment risk strategies. When investment policies are chosen, agency costs appear as a critical element in the model.

Further evidence of the agency motive for holding cash can be found in Dittmar et al. (2003), Pinkowitz et al. (2006), Dittmar and Mahrt-Smith (2007) and Harford et al. (2008). More recently, but still within the context of agency theory, Tran (2020) emphasized how external factors, such as the economic cycle, including the eventual financial crisis, affect cash holdings. The author found that the 2008 global financial crisis decreased the controlling effect of shareholder rights on corporate cash holdings, regardless of any control agency mechanism. Following a similar line of research, Tekin (2020) and Tekin et al. (2021) examined whether an agency cost explanation is valid for cash holdings during and after the financial crisis. During a financial crisis, agency costs tend to be higher than usual and the agency motive for holding cash is greater. The authors assessed the role of governance in cash management in 26 Asian developing countries and found that firms with poor governance increased their cash levels after the financial crisis. They concluded that cash holdings had a substitution effect on governance due to changes in managers’ risk aversion perceptions.

Cash management relates to financial constraints. The impact of financial restrictions on optimal cash holdings in the context of a financial crisis was considered by Tekin and Polat (2020), who compared firms in a highly regulated market with firms in a relatively unregulated market in the United Kingdom. The authors found that less-regulated firms had a faster adjustment of cash over the period 2002-2017. However, these firms decreased their cash adjustment speed more than highly regulated firms did during the financial crisis. Using a sample of firms from 26 developing Asian economies from 1991 to 2016, Tekin (2022) recently showed that financially constrained firms increased their cash levels more than financially unconstrained firms after the 2008 global financial crisis. In summary, exogenous shocks such as financial crises represent an important external factor in cash management.

Conversely, Foley et al. (2007) identify the tax motive for holding cash. More precisely, they found that the U.S. corporations, that would incur tax consequences associated with repatriating foreign earnings, hold higher levels of cash. Bates et al. (2009) showed that the average cash-to-assets ratio for U.S. industrial firms doubled from 1980 to 2006. They argue that the precautionary motive for cash holdings plays an important role in explaining the increase in cash ratios. From an analysis of the literature, Bates et al. (2009) summarized two additional motives for holding cash:

3. The agency motive, which is the need for cash derived from conflicts of interest among managers and owners.
4. The tax motive, which is the desire to avoid tax consequences associated with repatriation of foreign earnings.

Gao et al. (2013) analyzed a sample of public and private U.S. firms during the period 1995-2011 to conclude that public firms hold more cash than private firms. By examining the drivers of cash policies for each group, the authors attribute this difference to the much higher agency costs in public firms. Using a similar period (1998–2011), Pinkowitz et al. (2016) showed that U.S. firms held more cash on average than similar foreign firms. However, they argued that country characteristics had negligible explanatory power for the differences in cash holdings between U.S. firms and their foreign twins. Graham and Leary (2018) included the historic perspective in the analysis by studying average and aggregate cash holdings of companies in the U.S. from 1920 to 2014. Corporate cash holdings doubled in the first 25 years of the sample before returning to 1920 levels by 1970. Since then, the average and aggregate patterns have diverged.

Interest rates and environmental and health motives have recently been included in cash holding analyses. Gao et al. (2021) highlighted a non-monotonic relation between corporate cash and both real and nominal interest rates in both aggregate and firm-level data. The authors argue that these results imply that interest rates are unlikely to be the cause of the recent increase in corporate cash. Tan et al. (2021) compared cash holdings before and after the Environmental Inspection Program in China during the period 2014-2018 for manufacturing firms included and non-included in the program. The results suggest that this environmental program enhanced cash management efficiency because firms included in the program accumulated less cash. Finally, Alvarez and Argente (2022) focused on the impact of COVID-19 in household’s cash management behavior, considering the choice of means of payment and the average size and frequency of cash withdrawals. The authors used data on ATM (automated teller machine) cash disbursements in Argentina, Chile, and the U.S. to show that the intensity of the virus increased transaction costs.

## A multidimensional taxonomy of the cash management problem

Cash flow management concerns the efficient use of a company’s cash and short-term investments (Gregory 1976). Cash is then viewed as a stock, a buffer, such as an inventory of wheat or bolts. Holding cash has a cost because of it being idle but, at the same time, transferring idle money to alternative investments is also costly. How much money should companies keep to operate efficiently? Identifying an appropriate answer to this question is the main goal of CMP. However, several aspects and dimensions must be considered to establish the boundaries of the problem. Hereafter, we focus on the main dimensions of the cash management problem, defining a cash management problem taxonomy to classify past research and identify open research questions.

### Cash management models

In an attempt to solve CMP, several cash management models have been proposed to control cash balances based on a set of levels or bounds. CMP was first proposed from an inventory control perspective by Baumol (1952) in a deterministic manner. Later, Miller and Orr (1966) followed a stochastic approach, assuming that cash balance changes are random. Many other models have been developed based on these two seminal works. Most previous models assume a set of bounds to control cash balances; however, alternative configurations are also suitable.

### Cash flow process

Cash flow statistical characterization is also a key issue in understanding cash management. Separation between inflows and outflows, or receipts and disbursements, is the basic breakdown, but a more detailed separation can be helpful when trying to extract patterns from data. In this sense, Stone and Miller (1981, 1987) suggest the utility of problem structuring, or breaking down a problem into different subproblems, to appropriately handle cash flow forecasting as a key task in cash management. In addition, common assumptions on the statistical properties of cash flows include (i) normality, meaning that its values are centered around the average following a Gaussian distribution; (ii) independence, meaning that its values are not correlated with each other; and (iii) stationarity, meaning that its mean and variance are constant with time. However, little empirical evidence on the statistical properties of cash flow has been provided, with the exception of Mullins and Homonoff (1976), Emery (1981), Pindado and Vico (1996).

### Costs in cash management

The main objective of managing cash is to keep the amount of available cash as low as possible while still keeping the company operating efficiently. Additionally, companies may place idle cash in short-term investments (Ross et al. 2002). Thus, the cash management problem can be viewed as a trade-off between holding and transaction costs. On the one hand, holding costs are usually opportunity costs due to idle cash that can be allocated to alternative investments. Holding too much cash is inefficient but holding too little may result in high shortage costs. On the other hand, transaction costs are associated with the movement of cash from/into a cash account into/from any other short-term available asset, such as treasury bills and other marketable securities. In summary, if a company tries to keep balances too low, holding costs will be reduced, but undesirable situations of shortage will force the sale of available marketable securities, thereby increasing transaction costs. By contrast, if the balance is too high, low trading costs will be incurred due to unexpected cash flow, but the company will carry high holding costs because no interest is earned on cash. Therefore, the company must optimize its target cash balance.

### Desired objectives

In cash management literature, the focus is typically placed on a single objective, namely, cost. Except for Zopounidis (1999), Salas-Molina et al. (2018), cash management and multi-criteria decision-making are not usually linked concepts in financial literature. However, risk management is an important task in decision-making, and since different cash strategies entail different degrees of risk, a quantitative approach to measure risk is required. Furthermore, due to the different degrees of risk that firms are willing to accept, risk preferences are also an important issue for decision-makers.

### Solving the cash management problem

Cash management poses a general optimization problem, namely, determining a policy that optimizes objective functions. However, several different techniques have been used to solve this optimization problem, ranging from mathematical programming, such as dynamic programming (Eppen and Fama 1968; Penttinen 1991) and control theory methods Sethi and Thompson (1970), to approximate techniques such as genetic algorithms (Gormley and Meade 2007; da Costa Moraes and Nagano 2014). An important question regarding alternative solvers is the optimality of solutions, which is a desired objective, but must be balanced with computational and deployment costs.

### Managing multiple bank accounts

In cash management literature, cash management systems with multiple bank accounts have received little attention from the research community, with the exception of Baccarin (2009). However, cash management systems with multiple bank accounts are a rule, rather than an exception, in most firms.

Once the six main dimensions of the CMP are established, namely, models, cash flow, costs, objectives, solvers and number of accounts, we are in a position to review the most relevant cash management models proposed in the literature.

## A review of the main contributions to the cash management problem

Although the advancement of a specific research topic is gradual rather than sharp, the history of CMP is long enough to distinguish at least two main periods: the classical period up to 2000 and the modern period from 2000 onwards. Since the initial inventory approach to CMP by Baumol (1952), the classical period is characterized by the common two-assets framework, linear cost functions, and the minimization of cost as the single goal of cash managers. However, a multidimensional approach to CMP emerges with Baccarin (2009), who considered cash management systems with multiple bank accounts and non-linear cost functions. We argue that this change in perspective and implied complexity gives rise to a new period in the study of CMP. In the following sections, we present a review of the most relevant works on CMP from Baumol (1952) to Baccarin (2009) and consider the most recent contributions. We respect the authors’ notations and clarify issues regarding notation when necessary for comparison purposes.

### Baumol (1952)

**Fig. 1:**
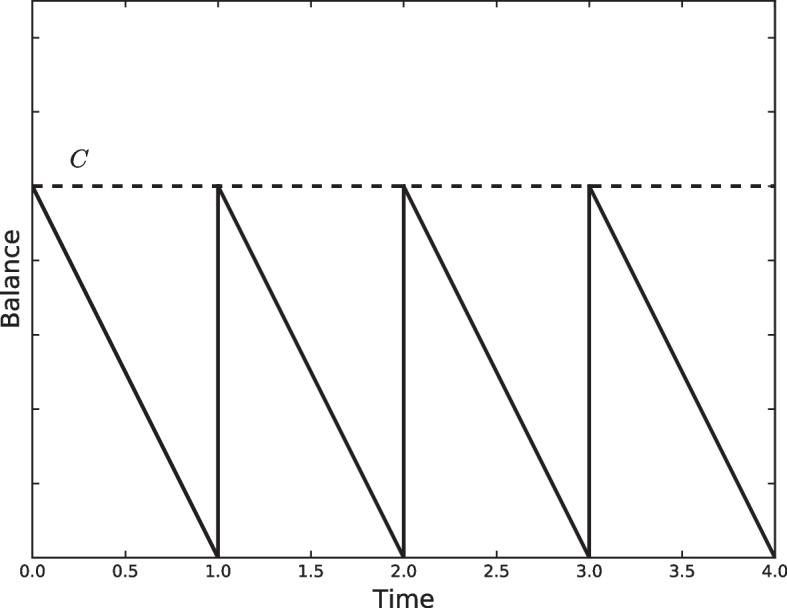
The Baumol model

The inventory control approach to the cash management problem was introduced by Baumol (1952). The author expected that inventory theory and monetary theory would learn from one another. However, several important assumptions were made to, using the exact Baumol’s words, abstract from precautionary and speculative demands. The most important was that transactions were perfectly foreseen and occurred in a steady stream. Baumol assumed that an outflow of *T* dollars occurred for a given period in a steady stream. To offset these outflows, inflows can be obtained by borrowing or withdrawing from an investment at a cost of *i* dollars per dollar per period. An additional assumption is made by considering that these withdrawals are performed in many *C* dollars, evenly spaced in time, with a fixed cost of *b* dollars (see Fig. 1).

Under these constraints, cash managers make *T*/*C* withdrawals for a given period, and the total cost is given by

1 $$\begin{aligned} \frac{bT}{C}+\frac{iC}{2} \end{aligned}$$

where the first part of the equation is the number of transactions multiplied by the unitary fixed cost of each transaction and the second part is the average cash balance multiplied by the cost of holding this balance. Then, the goal for cash managers is to choose *C* such that Eq. (1) is minimized. Setting the derivative of the total cost with respect to *C* to zero, we obtain the value of *C* that minimizes (1) as follows:

2 $$\begin{aligned} C=\sqrt{\frac{2bT}{i}}. \end{aligned}$$

The steady stream of payments and absence of receipts during the relevant period make this model impractical in many real applications. It was “only a suggestive oversimplification,” in the author’s own words. However, the first step in the inventory control approach to the cash management problem was performed. Interestingly, Baumol also envisioned the inherent task of forecasting cash flow by stating that with sufficient foresight, if receipts can meet payments, savings in the use of cash can be achieved.

Summarizing, Baumol (1952) initiated the inventory approach to the cash management problem proposing a deterministic model with uniform cash flows, with the objective of minimizing fixed transaction and holding costs for a single bank account using analytical methods.

### Tobin (1956)

Tobin argued that cash requirements depend inversely on the interest rate for a given volume of transactions, governed by the lack of synchronization of receipts and disbursements. The higher the lack of synchronization, the higher the need for transaction balances. However, there is no need to hold a cash balance. Instead, cash managers have the opportunity to maintain balances in assets with higher yields, such as bonds or marketable securities. When cash is needed, these assets could be shifted to cash again for payments. Consequently, it is likely that the amount of cash held for transaction purposes is inversely related to the interest rates of such alternative assets.

**Fig. 2:**
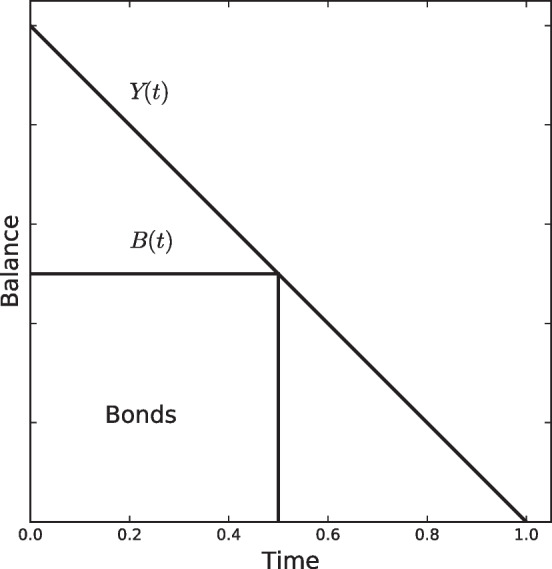
The Tobin model

Given an interest rate *r*, the problem is to find the relationship between what is held in cash and what is held in alternative assets to maximize interest earnings, net of transaction costs. At the beginning of each period $t=0$, an amount *Y* is held by the cash manager that is uniformly disbursed until the end of period $t=1$ when no cash is available, as shown in Fig. 2. Thus, the total transaction balance is $T(t)=Y(1-t)$ with $0 \ge t \ge 1$. However, this total *T*(*t*) can be divided between cash *C*(*t*) and bonds *B*(*t*) such that $T(t)=C(t)+B(t)$, where *B*(*t*) yields interest *r* per time period. Three different questions are then faced by Tobin: (i) given *r* and a fixed number *n* of transactions, determine the optimal timing and amounts to be held in cash and bonds; (ii) given *r* but a variable number *n* of transactions, determine the optimal $n^*$; and (iii) how does $n^*$ depends on *r*?

Considering transaction *x* between bonds and cash, the transaction cost is given by $a + b \cdot x$, with $a,b >0$. Then, for the general case, Tobin proves that the average number of bonds is given by

3 $$\begin{aligned} \overline{B} = \frac{n-1}{2n} Y \left( 1 - \frac{4b^2}{r^2} \right) \end{aligned}$$

where $n \ge 2$ and $r \ge 2b$. In order to determine the optimal number of transactions, the next profit function is maximized:

4 $$\begin{aligned} \pi _n = \frac{n-1}{2n} Y r \left( 1 - \frac{2b}{r} \right) ^2 - na \end{aligned}$$

that is a decreasing function of *n*. Then, the optimal number of transactions $n^*$ is greater than two when $1/12 Y r (1 - 2b/r)^2 \ge a$ holds true. Finally, the relationship between the optimal number of transactions $n^*$ and interest rate is given by Eq. (3). Since $B_n$ is an increasing function of *n*, and $n^*$ directly varies with *r*, the optimal proportion of bonds also directly varies with *r*; consequently, the proportion of cash inversely varies with *r* for sufficiently high rates.

Smith (1986) proposed a *Dynamic Baumol-Tobin Model of Money Demand*. However, this Baumol-Tobin model is more closely related to the Constantinides and Richard (1978) model than with the initial proposals by Baumol (1952) and Tobin (1956). More recently, Mierzejewski (2011) followed Tobin’s approach, according to which companies hold cash as a behavior towards risk, to propose a theoretical model of equilibrium in cash-balance markets, which is beyond the scope of this thesis.

Summarizing the above, the Tobin (1956) model is also a deterministic model dealing with a uniform cash flow such as the Baumol (1952) but incorporating the interest rate as a key parameter. In addition, Tobin considered not only fixed costs, but also variable transaction costs between two alternative assets, namely, bonds and cash. The goal was to minimize costs, and an analytical solution was provided.

### Miller and Orr (1966)

Miller and Orr introduced the stochastic cash balance problem by relying on the fact that the cash balance does not fluctuate steadily but rather irregularly for many companies, resulting in an impractical application of the Baumol model. Miller and Orr developed a simple model following an opposite approach to Baumol by considering stochastic cash flows. From a predictability point of view, Miller and Orr shifted from the perfect knowledge of cash flows in Baumol model to cash flows generated by a stationary random walk, from a deterministic approach to completely stochastic cash flows. They considered cash flows to be characterized as a sequence of independent and symmetric Bernoulli trials. They supposed that the cash balance will either increase or decrease by *m* dollars with probability $p=1/2$. The main features of this approach are independence, stationarity, zero-drift, and the absence of regular swings in cash flows. Moreover, they ignored shortages and variable transaction costs.

**Fig. 3:**
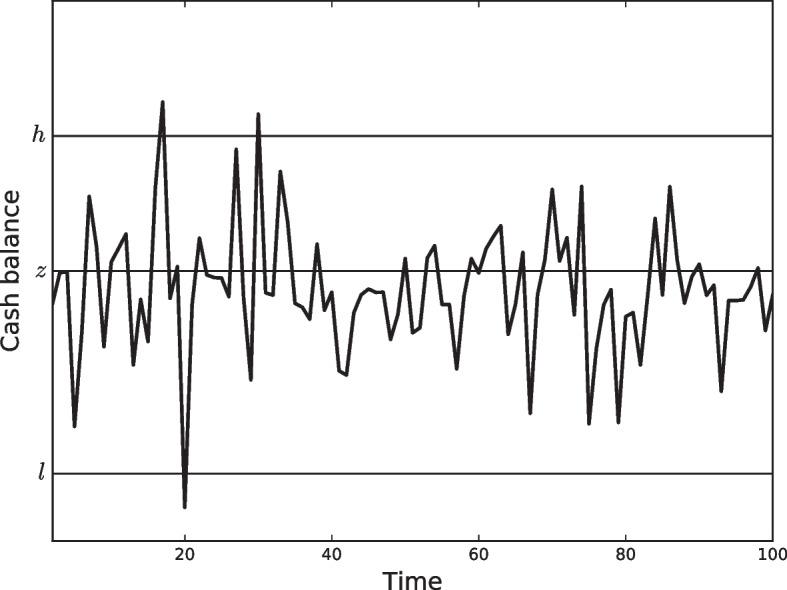
The Miler-Orr model

In their first attempt to deal with the corporate cash management problem, they assumed that companies seek to minimize the long-term average costs of managing the cash balance under a simple policy. This policy sets a lower bound, zero, and an upper bound, *h*, where cash balance is allowed to wander between the lower and upper levels. We say that the Miller and Orr (1966) is a Bound Based Model (BBM). Apart from the cash balance, the model also assumes the existence of a second asset of any kind, such as interest bearing assets or marketable securities grouped in a portfolio of investments that are easily transformed into cash at the company’s convenience. The policy implies that, when the upper bound reaches a withdrawal transfer, the balance is restored to a target level of *z*. Similarly, when the cash balance reaches zero, a positive transfer will be made to restore the balance to *z*, as shown in Fig. 3.

Although Miller and Orr set the lower limit to zero in their work, in practice, a real cash manager should set the lower limit above zero for precautionary motives. This lower limit represents a safety cash buffer, and its selection depends on the level of risk the company is willing to accept. This model variation can be found in (Ross et al. 2002), which sets a lower limit *l* and an upper bound *h*. When *h* is reached, a withdrawal transfer is performed to restore the balance to the target level of *z*. Similarly, when the cash balance reaches *l*, a positive transfer is made to restore the balance to *z*. Formally, the transfer occurring at time *t*, $x_t$, is elicited by comparing the current cash balance, $b_{t-1}$, with the lower and upper bounds:

5 $$\begin{aligned} x_t=\left\{ \begin{array}{lll}z-b_{t-1}, &{} \hbox {if} &{} b_{t-1} > h \\ 0, &{}\hbox {if} &{} l<b_{t-1}<h \\ z-b_{t-1}, &{} \hbox {if} &{} b_{t-1} < l \end{array}\right. \end{aligned}$$

To obtain the limits, once the cash manager sets the lower limit *l*, the optimal values of the policy parameters *h* and *z* are derived from the expected cost per day over any planning horizon of *T* days, given by

6 $$\begin{aligned} E(c)= \gamma \frac{E(N)}{T}+v E(M) \end{aligned}$$

where *E*(*c*) is the expected cost per day, *E*(*N*) is the expected number of transfers during the planning period *T*, $\gamma$ is the cost per transfer, *E*(*M*) is the average daily cash balance, and *v* is the daily interest rate earned on the portfolio as the opportunity cost of idle cash. By letting $Z=h-z$, the problem can be stated in terms of the variance of the net cash flow as:

7 $$\begin{aligned} \underset{Z,z}{\textrm{arg min}} \,E(c) = \underset{Z,z}{\textrm{arg min}} \,\frac{\gamma \sigma ^2}{Zz}+\frac{v(Z+2z)}{3} \end{aligned}$$

where the first part of the equation is the transfer cost term, and the second part is the holding cost term. The average cash balance is $(h+z)/3$. Hence, the optimal parameters are given by

8 $$\begin{aligned} z^{*}=\left( \frac{3\gamma \sigma ^2}{4v}\right) ^{1/3} \end{aligned}$$

and

9 $$\begin{aligned} Z^{*}=2z^{*}. \end{aligned}$$

or in terms of the original parameters

10 $$\begin{aligned} h^{*}=3z^{*}. \end{aligned}$$

The equivalent equations for the case of a lower bound (*l*) distinct from zero can easily be derived, as presented in Ross et al. (2002), to obtain

11 $$\begin{aligned} z^{*}=l+\left( \frac{3\gamma \sigma ^2}{4v}\right) ^{1/3} \end{aligned}$$

and

12 $$\begin{aligned} h^{*}=3z^{*}-2l. \end{aligned}$$

The major implication and main novelty of this model in comparison to the Baumol model is the presence of the observable variance of the net daily cash flow. As in the case of the Baumol model, the greater the transfer cost ($\gamma$), the higher the target cash balance (*z*), and the greater the daily interest rate (*v*), the lower the target cash balance (*z*). However, the greater the uncertainty of the net daily cash flow, measured by $\sigma ^2$, the higher the target cash balance (*z*), and the higher the difference between the lower bound (*l*) and the higher bound (*h*). This represents the first step towards a more practical approach to the corporate cash management problem because common sense shows that the greater the uncertainty, the greater the chance that the balance will drop below the lower bound.

Several extensions of the model have been considered to incorporate systematic drift in the cash balance and to allow for more than one portfolio asset with different transfers and holding costs. Despite the assumption of the totally stochastic mechanism of cash flow, the authors pointed out the presence of both stochastic and deterministic, or at least highly predictable, elements in cash flow, such as payroll disbursements or dividend payments. However, they argued that the gains from exploiting any cash flow patterns are by no means sufficiently large to offset the added costs of model development and implementation.

In summary, Miller and Orr (1966) was the first stochastic cash management model proposed in the literature. They introduced the concept of bounds or control limits, which are directly linked to the statistical properties of cash flows and are assumed to be random walks. Only fixed transaction costs were considered, and analytical solutions were provided for a single objective and cash account.

### Eppen and Fama (1969)

**Fig. 4:**
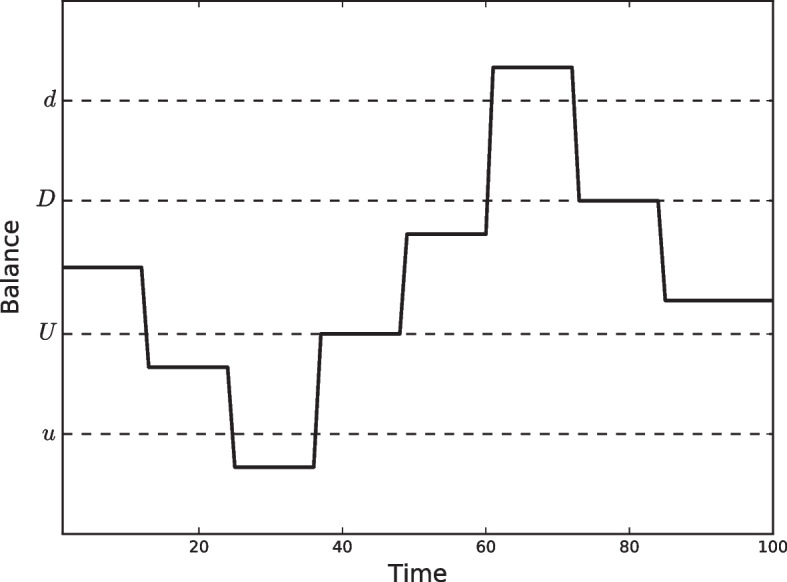
Eppen-Fama model representation with two return points

A variation of the Miller and Orr (1966) model was introduced by Eppen and Fama (1969) following a dynamic programming approach. However, it was a previous publication (Eppen and Fama 1968) which provided a complete analysis of the effect of variations in transfer, holding, and penalty costs on the optimal policies. The Eppen-Fama model is a generalization of the stochastic Miller-Orr model, in which transfer costs contain both fixed and variable components. They showed that if transfer costs have a fixed cost as well as a cost, proportional to the amount transferred, the optimal strategy is in the form of two limits (*u*, *d*) and two return points (*U*, *D*), one for each limit. In this model, when the cash balance reaches the upper bound (*d*), it is immediately restored to the upper return point (*D*), and when it reaches the lower bound (*u*), it is restored to the lower return point (*U*), as shown in Fig. 4.

Following the Markovian approach, they assumed that the probability mass function of the transitions between different possible states is known and stationary. This assumption implies the process of discretization of the cash balance. At any point in time, the cash balance can be in one of *N* possible states, $i=1,2,...N$, each representing a discrete level of cash balance. The lowest level occurs in state 1 and the highest in state *N*, and each successive level differs by some constant *R*, for example 1000 €.

For the general case, two cost functions are defined. First, the transfer cost ($t_{i}^{k}$) caused by moving the cash balance from state *i* to state *k*:

13 $$\begin{aligned} t_{i}^{k}=\left\{ \begin{array}{lll}K_{u}+c_{u}(k-i) &{} \hbox {if} &{} k>i; K_{u},c_{u}>0, \\ 0 &{} \hbox {if} &{} k=i, \\ K_{d}+c_{d}(i-k) &{} \hbox {if} &{} k<i; K_{d},c_{d}>0\end{array}\right. \end{aligned}$$

where $K_u$ and $c_u$ are the fixed and variable components of a positive cash movement, respectively, and $K_d$ and $c_d$ are the fixed and variable components of a negative cash movement, respectively. Second, the holding or penalty cost (*L*(*k*)) associated with starting a period in state *k* can be defined as follows:

14 $$\begin{aligned} L(k)=\left\{ \begin{array}{lll}c_{p}(M-k) &{} \hbox {if} &{} k<M;c_{p}>0, \\ c_{h}(k-M) &{} \hbox {if} &{} k>M;c_{h}>0\end{array}\right. \end{aligned}$$

where $c_p$ is the marginal penalty cost per period per *R* unit of cash, $c_h$ is the marginal holding cost per period per *R* unit of cash, say 1000 €, and *M* is the minimum cash balance that must be maintained because of any condition required by banks. In the absence of this restriction, *M* is usually set to zero as the minimum cash balance required to be held in the bank account.

Recall that Miller and Orr (1966) suggests the use of two or three bounds. To account for fixed and variable transaction costs, Eppen and Fama (1968) proposed the use of four bounds. From an experimental perspective, the authors pointed out that higher dispersion in the probability distribution caused the outer bounds *u* and *d* and the return points *U* and *D* to be further away from zero. Therefore, in practical applications, it is highly recommended to carefully estimate the probability distribution, particularly in extremes. Moreover, when both the probability distribution and cost function are symmetric about zero, the optimal policies are symmetrical.

In summary, several interesting contributions on the practical side of the corporate cash balance problem were made by Eppen and Fama under the assumption of cash flow following a random walk. They considered both fixed and variable transaction costs, resulting in a policy based on four bounds aimed at minimizing costs. They proposed linear programming as a solver in Eppen and Fama (1968) and dynamic programming in Eppen and Fama (1969) for a single cash bank account.

### Daellenbach (1971)

Daellenbach proposes an improvement to the Eppen and Fama (1969) model, claiming that his model is a generalization of the Eppen-Fama model to situations where bank account overdrafts are not possible, and using two different sources of short-term funds, namely, marketable securities and short-term loans. Furthermore, in contrast to previous models, the probability distribution of cash flows is not necessarily stationary and the length of the review period may vary from period to period. Again, a decision about the adjustment of the cash balance must be made; however, in this model, an allocation decision about either marketable securities or borrowing transactions is also necessary. A dynamic programming approach was proposed for labeling periods in the planning horizon as $n=N$ for the first period and $n=1$ for the last period. Three state variables were then considered to describe the cash balance situation:

1. $B_n$ or the cash balance at the beginning of period *n* carried forward from $n+1$.
2. $Z_n$ or the borrowing balance at the beginning of period *n* carried forward from $n+1$.
3. $S_n$ or the marketable securities balance at the beginning of period *n* carried forward from $n+1$.

If $X_n$ and $Y_n$ denote transactions in the form of borrowings or marketable securities, respectively, and $R_n$ is the sum of uncontrollable cash transactions in period *n* with the probability density function $f_n(r_n)$, the following balance equation is used to link period $n-1$ to period *n*:

15 $$\begin{aligned} B_{n-1}= & {} B_n+X_n+Y_n+R_n \end{aligned}$$

16 $$\begin{aligned} Z_{n-1}= & {} Z_n+X_n, \,\,\,\,Z_n\ge 0 \end{aligned}$$

17 $$\begin{aligned} S_{n-1}= & {} S_n-Y_n, \,\,\,\,S_n\ge 0 \end{aligned}$$

subject to:

18 $$\begin{aligned}{} & {} B_n+X_n+Y_n \ge 0 \end{aligned}$$

19 $$\begin{aligned}{} & {} Z_n+X_n \ge 0 \end{aligned}$$

20 $$\begin{aligned}{} & {} S_n-Y_n \ge 0 \end{aligned}$$

meaning that, (i) the initial cash balance before any adjustment has to be non-negative; (ii) the outstanding borrowing balance cannot be below zero; and (iii) marketable securities cannot be sold short.

According to the previous equations, the state variable set for the cash position at the beginning of period *n*, prior to any cash balance adjustment, is denoted by $\Omega _n=(B_n,Z_n,S_n)$, the decision variables are $(X_n,Y_n)$, the total cost is the sum of (i) fixed and variable transaction costs for borrowing, (ii) fixed and variable transaction costs for marketable securities, (iii) interest cost on borrowings, (iv) returns on marketable securities (note that this is a negative cost or a benefit), and (v) penalty costs for cash shortages. These costs can be summarized as follows:

21 $$\begin{aligned} \begin{aligned} T_n(X_n,Y_n;\Omega _n)=H_1(X_n)+H_2(Y_n)+c_{1n}(Z_n+X_n) \\ -c_{2n}(S_n-Y_n)+L_n(B_n+X_n+Y_n) \end{aligned} \end{aligned}$$

where $H_1(X_n)$ is the borrowing cost function computed as

22 $$\begin{aligned} H_1(X_n)=\left\{ \begin{array}{rrr}-b_{1}^{-}X_n &{} \hbox {if} &{} X_n<0, \\ b_{1}^{+}X_n &{} \hbox {if} &{} X_n \ge 0,\end{array}\right. \end{aligned}$$

where $b_{1}^{-}, b_{1}^{+}$ is the variable borrowing transaction costs for cash increases (+) and decreases (-), $H_2(Y_n)$ is the marketable securities cost function computed as

23 $$\begin{aligned} H_2(Y_n)=\left\{ \begin{array}{rrr}-b_{2}^{-}Y_n &{} \hbox {if} &{} Y_n<0, \\ b_{2}^{+}Y_n &{} \hbox {if} &{} Y_n \ge 0,\end{array}\right. \end{aligned}$$

where $b_{2}^{-}, b_{2}^{+}$ are variable marketable security transaction costs for cash increases (+) and decreases (-), respectively; $c_{1n}$ is the interest cost on ending loan balances; $c_{2n}$ is the return on ending marketable securities holdings; $L_n(B_n)$ is the expected cost of cash shortage incurred at the end of period *n* computed as:

24 $$\begin{aligned} L_n(B_n+X_n+Y_n)=c_{3n}\int _{-\infty }^{-(B_n+X_n+Y_n)}(B_n+X_n+Y_n+r_n)f_n(r_n)dr_n \end{aligned}$$

where $c_{3n}$ is the penalty for negative ending cash balances in period *n*.

Considering alternative funding sources, such as borrowings and marketable securities, introduces additional considerations on priorities based on feasible permutations of the cost coefficients as follows:

- Case 1. If $-b_{2}^{-}+c_2 \le -b_{1}^{-}+c_1 \le b_{1}^{+}+c_1 \le b_{2}^{+}+c_2$, then borrowing transactions are preferred over marketable securities.
- Case 2. If $-b_{1}^{-}+c_1 \le -b_{2}^{-}+c_2 \le b_{2}^{+}+c_2 \le b_{1}^{+}+c_1$, then marketable security transactions are preferred over borrowing.
- Case 3. If $-b_{2}^{-}+c_2 \le -b_{1}^{-}+c_1 \le b_{2}^{+}+c_2 \le b_{1}^{+}+c_1$, then borrowing transactions are preferred over marketable securities for cash withdrawals, and marketable securities are preferred over borrowing for cash procurements.
- Case 4. If $-b_{1}^{-}+c_1 \le -b_{2}^{-}+c_2 \le b_{1}^{+}+c_1 \le b_{2}^{+}+c_2$, then marketable securities are preferred over borrowing transactions for cash withdrawals, and borrowings are preferred over marketable securities for cash procurements.
- Case 5. If $-b_{2}^{-}+c_2 \le b_{2}^{+}+c_2 \le -b_{1}^{-}+c_1 \le b_{1}^{+}+c_1$, then borrowing transactions are preferred over marketable securities for cash withdrawals, and marketable securities are preferred over borrowings for cash procurements.

As a result, the Daellenbach model can be regarded as an extension of the Eppen and Fama (1968, 1969) model, but with four return points: $\{U_{1n},D_{1n}\}$ denote the use of borrowings as the source of funds, and $\{U_{2n},D_{2n}\}$ denote the use of marketable securities as the source of funds. The optimal policy gives preference to the source of funds dictated by the previous five cases based on the cost coefficients. If either constraint (19) or (20) prevents the completion of the transaction, then use the return point relevant to the other source of funds.

Subsequently, Daellenbach (1974) pointed out an important issue by posing the following general question: *Are cash management models worthwhile?* The objective was to determine the upper bounds of potential savings that could be realized by applying cash management models. In this study, a variant of the model in Daellenbach (1971) is proposed to consider fixed and variable transaction costs. In addition, a deterministic shortage cost function that charges negative cash balances at the end of the day is defined instead of the previous stochastic one. The main criticism of cash management models is based on the assumption of perfectly predictable cash flows. Any cost estimate based on perfect predictions will provide optimistic lower bounds for the actual cost incurred, which corresponds to determining what the optimal policy would have been given the actual cash flow. Using random normal simulations, the author estimated the upper bounds obtained by this variant of his cash management model on the performance of a hypothetical cash manager. The author concluded that the benefits of cash management optimization models were, in most cases, highly uncertain and offered a very small economic return.

In summary, Daellenbach (1971) used dynamic programming to provide a solution to the CMP as a set of control bounds but considered two available sources of funds, namely, marketable securities and short-term loans. In addition, the usual assumption on stationary cash flow was relaxed and fixed and variable transaction costs were considered as objectives to minimize.

### Stone (1972)

*The use of forecasts and smoothing in control-limit models for cash management* was proposed by Stone (1972). In this work, Stone first reviewed the assumptions of the Baumol (1952) and Miller and Orr (1966) models and pointed out a series of limitations of these models in real-world cash management situations. Stone argued that cash flows are neither completely certain, uniform, and continuous (as they are in the Baumol model) nor completely unpredictable (as they are in the Miller-Orr model). Most firms can forecast their cash flows. This is the first time that the concept of forecasting cash flows has been a key input to any cash management model. The author focused on the generally attempted tasks performed by cash managers. They usually:

1. Look ahead when buying and selling securities to incorporate data from their cash forecasts.
2. Smoothen cash flows by coordinating security maturities with predicted cash needs.
3. Buy the highest yielding securities subject to portfolio and liquidity constraints.
4. Maintain cash balances sufficient to meet banking requirements.

From these tasks, Stone derived the idea of including both forecasts and maturing securities in his model. The operation of this control-limit model is based on the ability to buy and sell securities of different maturities to reduce transaction costs by smoothing cash flows and thereby reducing the number of transactions. It is assumed that the current cash balance, $CB_0$, is known, and that a forecast of the net cash flow, $E(C_t)$, that will occur on each day *t* over the next *k* days is available. The expected level of cash balances *k* days from now is the sum of the current level of cash balances and the sum of *k* daily net cash flow. This can be expressed as

25 $$\begin{aligned} E(CB_k)=CB_0+\sum _{t=1}^{k}E(C_t). \end{aligned}$$

Alternatively, if the sum of net cash flows over the next *k* days is lumped into a single figure, the last equation can be rewritten as:

26 $$\begin{aligned} E(CB_k)=CB_0+E(SC_k). \end{aligned}$$

**Fig. 5:**
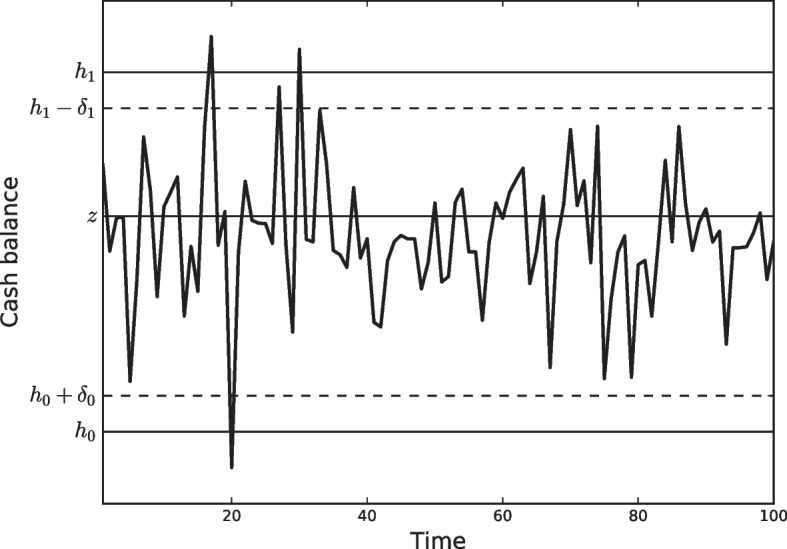
Structure of the Stone model with two sets of limits

Next, a number of simple rules are proposed to be followed by cash managers to return to the desired target balance *TB*, based on two sets of control limits as shown in Fig. 5. One set is defined by $h_1$ and $h_0$ as the upper and lower control limits for initiating considerations of a transactions. The other set is defined by $h_1-\delta _1$ and $h_0+\delta _0$ as the upper and lower limits, respectively, and determine if a transaction will actually be made.

The set of rules followed by cash managers to operate the model are summarized as follows.

1. If the current cash balance $CB_0$ is inside the control limits defined by $h_1$ and $h_0$, no action is taken.
2. If the control limits $h_1$ and $h_0$ are exceeded, the forecasts over the next *k* days is considered to decide whether a transaction should be made.
  - If the expected cash balance in the next *k* days, $E(CB_k)$, exceed the control limits defined by $h_1-\delta _1$ and $h_0+\delta _0$, a transaction is made to return the expected cash balance to the target level *TB* in *k* days.
  - No action is taken otherwise.

The innovation introduced by the Stone model is that when a transaction is made, the model returns the expected level of balance to the target level in *k* days rather than immediately returning the current balance to the target. Furthermore, the actual cash balance is the target plus the net cumulative forecast error. As $K_t$ is the number of transactions to be made, these rules can be represented mathematically as follows:

27 $$\begin{aligned} K_t=\left\{ \begin{array}{lll}TB-CB_0-E(SC_k), &{} \hbox {if} &{} CB_0> h_1\, \hbox {and}\, CB_0+E(SC_k)>h_1-\delta _1, \\ 0, &{}\hbox {if} &{} h_0<CB_0<h_1 \\ TB-CB_0-E(SC_k), &{} \hbox {if} &{} CB_0< h_0 \,\hbox {and}\, CB_0+E(SC_k)<h_0+\delta _0.\end{array}\right. \end{aligned}$$

Since the cash policy is fixed for a period of k-days, the use of forecasts forces the cash manager to monitor errors for *k* days after a transaction has occurred. However, the impact of the predictive accuracy of the forecasts on the policy performance was not evaluated. It is expected that a better prediction will lead to better policies, as hypothesized in Gormley and Meade (2007), and consequently, an evaluation of the impact of predictive accuracy is a mandatory step. Furthermore, efforts to improve predictive accuracy have associated costs that must be compared to the savings obtained to decide if further efforts are worthy. The impact of cash flow forecasts is an ongoing issue in cash management, which we address in Question 1, as we consider it a crucial challenge.

For the selection of the model parameters, no particular procedure was specified by Stone, although some suggestions were made, namely, not to treat them as fixed parameters, but rather adjust them as necessary. Simulation and the practitioner’s judgment were suggested as the best approaches to parameterization. The involvement of cash managers in the process of parameter selection was considered an advantage of this method. An alternative approach to deal with cash flow uncertainty was followed by Hinderer and Waldmann (2001) who developed a rigorous mathematical framework to include varying environmental factors in the cash manager decision-making process.

In summary, Stone was the first to formally develop a cash management model using forecasts as a key input. Consequently, they assume that cash flows are predictable to some extent. Several studies on daily cash flow prediction (Stone and Wood 1977; Stone and Miller 1981; Miller and Stone 1985; Stone and Miller 1987) represent an important contribution to cash management literature. However, the lack of a formal procedure to determine the set of parameters (bounds) of the look-ahead procedure, rather than the mere suggestion of using simulations, has become a serious limitation. No cost function was considered by Stone.

### Constantinides and Richard (1978)

Although Neave (1970) showed that the Eppen and Fama (1969) model was not optimal, Constantinides and Richard (1978) proved the existence of optimal simple policies for discounted costs when net cash flow followed a Wiener process. They studied the case of fixed and variable transaction costs and linear holding and penalty costs and used impulse control techniques to find sufficient conditions for an optimal policy defined by parameters $d \le D \le U \le u$. Similar to other bound-based models, control actions are only taken whenever the cash level either rises above *u* or falls below *d* money units.

Instead of the discrete time framework considered in Eppen and Fama (1968), Eppen and Fama (1969), Girgis (1968), Neave (1970), Constantinides and Richard assumed that decisions are made continuously over time. Moreover, they assumed that demand over any length of time is generated by a Wiener process, meaning that it is normally distributed with both the mean and standard deviation proportional to the length of time considered. However, they followed the impulse control approach of Bensoussan and Lions (1975) which was later extended by Richard (1977). This control technique is based on control actions taken at stochastic stopping times.

The problem formulation was similar to that used in previous studies on cash management. The cash balance at time *t* is defined as $x=x(t)$ and it is charged with a holding/penalty cost $C(x)={\text {max}} \{hx,-px\}$, with $h,p>0$. The transaction cost of changing the cash level from $x_0$ to $x_1$ is

28 $$\begin{aligned} B(x_1-x_0)=\left\{ \begin{array}{lll}K^+ + k^+(x_1-x_0) &{} \hbox {if} &{} x_1 \le x_0,\\ K^- + k^-(x_0-x_1) &{} \hbox {if} &{} x_1<x_0,\end{array}\right. \end{aligned}$$

with $k^+, k^-, K^+, K^- >0$, such that a zero-control action incurs a fixed cost.

In addition, the cumulative demand for cash in interval [*t*, *s*], denoted by *D*(*t*, *s*), is independent and normally distributed with mean $E[D(t,s)]=\mu (s-t)$ and variance ${\text {var}}[D(t,s)]=\sigma ^2(s-t)$, where $\mu$ and $\sigma ^2$ are constants. Thus, the cumulative demand is given by

29 $$\begin{aligned} D(t,s)=\mu (s-t)+\sigma (w(s)-w(t)) \end{aligned}$$

Where, *w* is a Wiener process in $\mathbb {R}$ with zero drift and a diffusion coefficient of one. However, the use of diffusion processes to represent the cash holding evolution is not new (Miller and Orr 1966).

In this framework, cash managers continuously observe cash levels and perform control actions when necessary. At any stopping time $\tau _i$, the applied control $\phi _i$, is a random variable that is independent of the future state of the system. An impulse control policy *v* is represented as a sequence of stopping times and controls, $v=[\tau _1,\phi _1; \tau _2,\phi _2; \ldots ]$. If $x(\tau _i^-)$ denotes the cash level at the stopping time $\tau _i$ before the control action $\phi _i$ is applied, and $x(\tau _i)$ denotes the cash level after the control action, then the state equations of the cash level when policy *v* is applied are given by

30 $$\begin{aligned} dx(t)=-\mu dt - \sigma dw(t) \end{aligned}$$

when $0 \le t < \tau _i$, with $x(0^-)=x_0$, and:

31 $$\begin{aligned} x(\tau _i)=x(\tau _i^-) + \phi _i, \,\,\,\, dx(t)=-\mu dt - \sigma dw(t) \end{aligned}$$

when $\tau _i \le t < \tau _{i+1}^-$, with $i \ge 1$. Given a policy *v* and an initial cash balance $x(0^-)=x_0$, the expected total cost from time zero to infinity, discounted to time zero, is

32 $$\begin{aligned} J_{x_0}(v)=E\left[ \sum _{i=1}^\infty e^{-\beta \tau _i} B(\phi _i)+\int _0^\infty e^{-\beta s} C(x(s))ds \right] \end{aligned}$$

where $\beta$ denotes the discount rate. The final goal is to choose policy $v^*$ such that $J_{x_0}(v^*) \le J_{x_0}(v)$, $\forall v \in \Omega$, where $\Omega$ is the class of all impulse control policies.

Let $V(x)=J_{x}(v)$ be the expected total cost from time *t* to infinity discounted to time *t* and conditional on the cash level $x(t^-)=x$. Note also that $V(x)\ge 0$ since all costs are non-negative. There are only two possible alternatives for cash managers: taking no control action or making the most convenient transaction in terms of future costs. By applying dynamic programming and assuming that the subsequent decisions are also optimal, *V*(*x*) must satisfy

33 $$\begin{aligned} V(x(t^-))={\text {min}}\left\{ \begin{array}{l}{\text {inf}}_{\xi } [B(\xi )+ E(C(x(t))dt+e^{-\beta dt}V(x(t)+dx))],\\ E(C(x(t))dt+e^{-\beta dt}V(x(t)+dx)).\end{array}\right. \end{aligned}$$

From this, the following theorem is derived.

#### Theorem 1

Suppose that $h>\beta k^-$ and $p>\beta k^+$ hold true, then , an optimal policy exists for the cash management problem. This policy is simple and is given by

34 $$\begin{aligned} y(x)=\left\{ \begin{array}{lll}D &{} \hbox {if} &{} x \le d,\\ x &{} \hbox {if} &{} d<x<u,\\ U &{} \hbox {if} &{} u \le x,\end{array}\right. \end{aligned}$$

Note that the previous theorem implies that, if $h<\beta k^-$, it will never be optimal to reduce the cash level as long as $K^->0$. Similarly, if $p<\beta k^+$, it will never be optimal to increase the cash level, as long as $K^+>0$. If both conditions, $h<\beta k^-$ and $p<\beta k^+$ hold, the optimal policy prescribes no intervention. In the special case of $h<\beta k^-$ and $p>\beta k^+$, it is optimal to increase the cash level, but not optimal to decrease the cash level. They then deal with an inventory problem in which the control action $\xi (x)$ is constrained to be non-negative.

This model was later extended to the case of quadratic holding-penalty costs in Baccarin (2002) and to a multidimensional cash management system and general cost functions in Baccarin (2009), when cash balances fluctuate as a diffusion process. Premachandra (2004) also used a diffusion process to propose a more generalized version of the Miller-Orr model which relaxes most of its restrictive assumptions. The Wiener process is also a diffusion process (Itô 1974).

In summary, in addition to considering continuous cash flows, the most important contribution of the Constantinides and Richard (1978) model is Theorem 1, which provides the necessary conditions to avoid the triviality of the cash policy. Furthermore, it represents the origin of several recent studies (Baccarin 2002; Premachandra 2004; Baccarin 2009) on cash management. However, the strong assumption of modeling cash flows as a diffusion process represents a serious limitation when dealing with empirical non-Gaussian cash flows.

### Penttinen (1991)

Penttinen presented myopic and stationary solutions for linear costs using a logistic distribution as the probability density function of random cash demand. Myopic one-period solutions have been suggested to avoid computational difficulties in multi-period applications with a large number of discrete states. In contrast to Constantinides and Richard (1978), Penttinen chose a discrete time framework because common planning and control practices in most organizations are typically performed in discrete intervals.

His main goal was to analyze the amount of suboptimality in myopic solutions. Thus, the problem formulation considers a stochastic cash balance in which demand $\delta$ is a random variable. The amount of cash at the beginning of each period *n* is denoted by *x* and the cash balance after a control action is taken is denoted by *y*(*x*). The author considers the transaction costs $a_n(y-x)$ as

35 $$\begin{aligned} a_n(y-x)=\left\{ \begin{array}{lll}K_n+k_n \cdot (y-x) &{} \hbox {if} &{} y-x>0, \\ 0 &{} \hbox {if} &{} y=x, \\ Q_n+q_n \cdot (x-y) &{} \hbox {if} &{} y-x<0,\end{array}\right. \end{aligned}$$

where $K_n,Q_n,k_n,q_n \ge 0$. In addition, the retained and penalty costs $m_n(y)$ charge the cash level *y* at the beginning of each period according to

36 $$\begin{aligned} a_n(y-x)=\left\{ \begin{array}{lll}r_n(y) &{} \hbox {if} &{} y>0,\\ p_n(-y) &{} \hbox {if} &{} y \le 0.\end{array}\right. \end{aligned}$$

Finally, the holding and shortage costs $l_n(z)$ charge the cash level z at the end of each period. Here, the amount of cash remaining is given by $z = y - \delta$ and the optimal balance at this point is zero because any positive balance is subject to a holding cost and any negative balance to a shortage cost:

37 $$\begin{aligned} l_n(z)=\left\{ \begin{array}{lll}h_n(z) &{} \hbox {if} &{} z>0,\\ s_n(-z) &{} \hbox {if} &{} z \le 0.\end{array}\right. \end{aligned}$$

The expected holding and shortage costs are given by the following loss function:

38 $$\begin{aligned} L_n(y)=\int _{-\infty }^\infty l_n(y-\delta )\phi _n(\delta )d\delta \end{aligned}$$

which is the convolution of $l_n(y-\delta )$ with the probability density function $\phi _n(\delta )$. Then, the optimal discounted value of future costs at the beginning of period *n* is:

39 $$\begin{aligned} C_n(x)= \underset{y}{{\text {inf}}} \lbrace a_n(y-x)+m_n(y)+L_n(y)+\alpha \phi _n * C_{n+1}(y)\rbrace \end{aligned}$$

where $\alpha$ is a discount factor, and $*$ denotes convolution. Note that, when $\alpha =0$, the dynamic model is called a myopic model. The optimal policy of this general convex model is given by

40 $$\begin{aligned} L^\prime (T)= & {} -k - m^\prime (T) \end{aligned}$$

41 $$\begin{aligned} L^\prime (U)= & {} q - m^\prime (U) \end{aligned}$$

42 $$\begin{aligned} L(t) - L(T)= & {} K + k(T-t) + m(T)- m(T) \end{aligned}$$

43 $$\begin{aligned} L(u) - L(U)= & {} Q + q(u-U) + m(U)- m(u) \end{aligned}$$

where $t \le T \le U \le u$ defines a transaction rule in the form of a simple policy $y_n(x)$ such that

44 $$\begin{aligned} y_n(x)=\left\{ \begin{array}{lll}T_n &{} \hbox {if} &{} x<t_n, \\ x &{} \hbox {if} &{} t_n \le x \le u_n, \\ U_n &{} \hbox {if} &{} x>u_n.\end{array}\right. \end{aligned}$$

Penttinen introduced logistic distribution to ease calculations. In this case, the optimal myopic policy is given by

45 $$\begin{aligned} T= & {} \mu + \frac{\ln [-(k+r-s)/(k+r+h)]}{d} \end{aligned}$$

46 $$\begin{aligned} U= & {} \mu + \frac{\ln [(q-r+s)/(-q+r+h)]}{d}. \end{aligned}$$

The reorder point *t* and disposal point *u* are derived numerically from *T* and *U* from Eqs. (42) and (43). To this end, an iterative procedure is presented to compute solutions that are expected to achieve rapid convergence. Different empirical results show the proportionality of policy parameters *t*, *T*, *U*, and *u* with the shortage cost ratio; thus, the higher the shortage cost, the higher the reorder and disposal points.

In contrast, stationary solutions are based on the assumption that each period possesses the same cost functions, and that cash demand is independent and identically distributed. Then, Penttinen presented additional empirical results on the amount of suboptimality between myopic and stationary solutions in the case of no fixed costs. His results show that the stationary model leads to slightly more cautious ordering policies.

In summary, it is important to highlight the assumption of the logistic distribution within the commonly used family of Gaussian cash flows to better represent empirical cash flows. Penttinen also assumed fixed and linear transaction costs to derive, by dynamic programming, two kinds of optimal policies, namely, myopic (minimizing short-term costs) and stationary (minimizing long-term costs). He considered both a single objective and single bank account in this proposal.

### Gormley and Meade (2007)

Gormley and Meade claimed *the utility of cash flow forecasts in the management of corporate cash balances* and proposed a Dynamic Simple Policy (DSP) to demonstrate that savings can be obtained using cash flow forecasts. They suggested the use of an autoregressive model as a key input for their model. However, gains in the forecast accuracy over the naive model are scant. Gormley and Meade expected that savings from using a non-naive forecasting model would increase if there were more systematic variations in cash flow and, consequently, higher forecast accuracy. If this hypothesis is correct, then the savings produced by a better forecasting model are expected to be significantly higher than those obtained by the naive forecasting model.

**Fig. 6:**
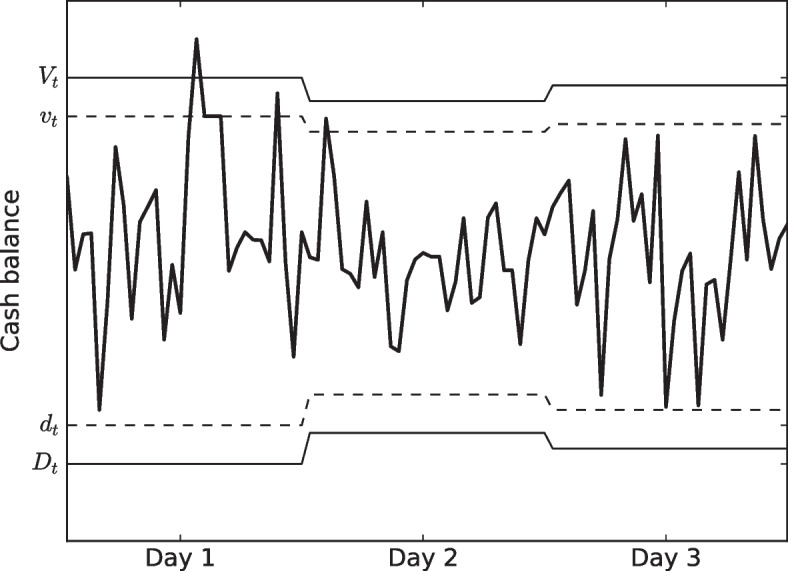
The Dynamic Simple Policy of Gormley-Meade

In their approach to the corporate cash management problem, Gormley and Meade used an inventory control stochastic model in which cash balances were allowed to move freely between two limits, as shown in Fig. 6: the lower (*D*) and the upper balance limit (*V*). When the cash balance reaches any of these limits, a cash transfer returns to the corresponding rebalance level (*d*, *v*), as shown in Fig. 6. Thus, the management of the cash balance over a period *T* is determined by a set of policy parameters or limits for the instant *t* that can be extended $\tau$ days ahead: $D_{t+\tau }$ is the lower balance limit at time $t+\tau$, $V_{t+\tau }$ is the upper balance limit at time $t+\tau$, $d_{t+\tau }$ is the lower rebalance level at time $t+\tau$, and $v_{t+\tau }$ is the upper rebalance level at time $t+\tau$.

The transfers for any prediction horizon are determined by

47 $$\begin{aligned} K_{t+\tau }=\left\{ \begin{array}{lll}v_{t+\tau }-\tilde{O}_{t+\tau -1}-\hat{w}_{t+\tau |t}, &{} \hbox {if} &{}\tilde{O}_{t+\tau -1}+\hat{w}_{t+\tau |t}>V_{t+\tau }, \\ 0, &{} &{}\hbox {otherwise,} \\ d_{t+\tau }-\tilde{O}_{t+\tau -1}-\hat{w}_{t+\tau |t}, &{} \hbox {if} &{} \tilde{O}_{t+\tau -1}+\hat{w}_{t+\tau |t}<D_{t+\tau }\end{array}\right. \end{aligned}$$

where $\tilde{O}_{t+\tau -1}$ is the predicted opening balance at time $t+\tau -1$, $\hat{w}_{t+\tau |t}$ is the predicted cash flow for $t+\tau$ using a model that has been trained up to time *t*. In this model, $D_{t+\tau } \le d_{t+\tau } \le v_{t+\tau } \le V_{t+\tau }$ and the following continuity function holds:

48 $$\begin{aligned} \tilde{O}_{t+\tau } = \tilde{O}_{t+\tau -1}+K_{t+\tau }+\tilde{\varepsilon }_{t+\tau |t} \end{aligned}$$

The expected cost over horizon *T* is given by the following objective function:

49 $$\begin{aligned} Cost=\sum _{\tau =1}^{T}\Gamma (K_{t+\tau })+\tilde{O}_{t+\tau }(h\cdot I_{\tilde{O}_{t+\tau }>0}+u\cdot I_{\tilde{O}_{t+\tau }<0}) \end{aligned}$$

where the transfer cost function $\Gamma$ is defined as

50 $$\begin{aligned} \Gamma (K_{t+\tau })=\left\{ \begin{array}{lll}\gamma _{0}^{-}-\gamma _{1}^{-}K_t &{} \hbox {if} &{} K_t<0, \\ 0 &{} \hbox {if} &{} K_t=0, \\ \gamma _{0}^{+}+\gamma _{1}^{+}K_t &{} \hbox {if} &{} K_t>0.\end{array}\right. \end{aligned}$$

The notation used by the expected and transfer cost functions is as follows: *h* is the holding cost per money unit of a positive cash balance at the end of the day; *u* is the shortage cost per money unit of a negative cash balance at the end of the day; $\gamma _{0}^{+}$ is the fixed cost of transfer into account;$\gamma _{0}^{-}$ is the fixed cost of transfer from account; $\gamma _{1}^{+}$ is the variable cost of transfer into account; $\gamma _{1}^{-}$ is the variable cost of transfer from account; $I_{\tilde{O}_{t+\tau }>0}$ is a boolean variable that equals one if $\tilde{O}_{t+\tau }>0$ is true, zero otherwise; $I_{\tilde{O}_{t+\tau }<0}$ is a boolean variable that equals one if $\tilde{O}_{t+\tau }<0$ is true, zero otherwise.

The authors used genetic algorithms to solve the CMP, that is, to estimate the parameters $\{D_{t+\tau }, d_{t+\tau }, v_{t+\tau }, V_{t+\tau }\}$ from $\tau =1, \ldots , T$. Moreover, because the model accepts forecasts as its main input, a cash flow autoregressive forecasting model was developed. To this end, a Box-Cox transformation (Box and Cox 1964) was used to achieve the normality of the real cash flow dataset used in this study.

In summary, Gormley and Meade (2007) proposed a cash management model that uses forecasts as a key input. Surprisingly, they did not refer to the work by Stone (1972) on the use of forecasts in cash management. They proposed evolutionary algorithms to derive cash policies within the usual context of fixed and linear transaction costs and a single objective. This solving procedure was recently followed in da Costa Moraes and Nagano (2014).

### Chen and Simchi-Levi (2009)

The concept of K-convexity was first used by Neave (1970) to show that the Eppen and Fama (1969) model may not be optimal. When fixed costs exist for both inflows and outflows, Chen and Simchi-Levi (2009) used the concept of (K,Q)-convexity by Ye and Duenyas (2007) to characterize the optimal policy in the stochastic cash balance problem. Their approach was closely related to inventory control, in that they used common inventory terminology rather than that usually employed in cash management research. For example, they speak about order and return rather than increase or decrease in cash transactions.

They considered a general cost function with holding and transaction costs. A transaction decision must be made at the beginning of each period. Let *x* be the cash balance at the beginning of period *n* before a decision is made and let *y* be the cash balance after a transaction is made. Transaction cost is computed as follows:

51 $$\begin{aligned} c(x,y)=\left\{ \begin{array}{lll}K+k(y-x) &{} \hbox {if} &{} y>x, \\ 0 &{} \hbox {if} &{} y=x, \\ Q+q(x-y) &{} \hbox {if} &{} y<x.\end{array}\right. \end{aligned}$$

where $K\ge 0$, $Q\ge 0$, and $k+q\ge 0$, assuming that $k\ge q$; that is, the positive variable transaction cost is greater than or equal to the negative variable transaction cost.

In contrast, the holding cost in time period *n* is described as a general cost function $l_n(z)$, which depends on the inventory level at the end of day *z* which, in turn, depends on the stochastic cash flow $\xi _n$. Therefore, the expected holding or penalty cost in period *n* is given by

52 $$\begin{aligned} L_n(y) = E[l_n(z)] = E[l_n(y-\xi _n)] \end{aligned}$$

In this study, the stochastic cash balance problem is formulated as a dynamic program, where $C_n(x)$ is the cost-to-go function at the beginning of a period when there are *n* periods left in the planning horizon, and the initial inventory level is *x*:

53 $$\begin{aligned} C_n(x)= \min _y\{c(y,x)+L_n(y)+\gamma E[C_{n-1}(y-\xi _n)]\} \end{aligned}$$

where $\gamma \in (0,1]$ denotes the discount factor.

They built a process to obtain the optimal policy based on the concept of (*K*, *Q*)-convexity (Ye and Duenyas 2007) of the recursive function $C_n(x)$. A real value function is called (*K*, *Q*)-convex for $K,Q \ge 0$. If for any $x_0$, $x_1$ with $x_0 \le x_1$ and $\lambda \in [0,1]$, the following condition holds:

54 $$\begin{aligned} f((1-\lambda )x_0+\lambda x_1)\le & {} (1-\lambda )f(x_0)+\lambda f(x_1)\nonumber \\{} & {} + \lambda K + (1-\lambda )Q-{\text {min}}\{\lambda ,1-\lambda \}{\text {min}}\{K,Q\}. \end{aligned}$$

We refer the interested reader to Chen and Simchi-Levi (2009) for further details on the properties of (*K*, *Q*)-convex functions and for proof that the cost-to-go function $C_n(x)$ is a (*K*, *Q*)-convex function. However, several additional definitions are required to derive the optimal policy.

55 $$\begin{aligned}{} & {} H_n(x)=L_n(x)+\gamma E[C_{n-1}(x-\xi _n)] \end{aligned}$$

56 $$\begin{aligned}{} & {} T_n \in {\text {argmin}}_x \{kx+H_n(x)\} \end{aligned}$$

57 $$\begin{aligned}{} & {} t_n = {\text {min}}\{x|kx+H_n(x)=K+kT_n+H_n(T_n)\} \end{aligned}$$

58 $$\begin{aligned}{} & {} t'_n = {\text {min}}\{x|kx+H_n(x)=K-Q+kT_n+H_n(T_n)\} \end{aligned}$$

59 $$\begin{aligned}{} & {} U_n \in {\text {argmin}}_x \{-qx+H_n(x)\} \end{aligned}$$

60 $$\begin{aligned}{} & {} u_n = {\text {max}}\{x|-qx+H_n(x)=Q-qU_n+H_n(U_n)\} \end{aligned}$$

61 $$\begin{aligned}{} & {} u'_n = {\text {min}}\{x|-qx+H_n(x)=K-Q-qU_n+H_n(U_n)\} \end{aligned}$$

where $t_n \le t'_n \le T_n$ and $u'_n \le U_n \le u_n$. Based on the previous definitions and assuming $K>Q \ge 0$, it is optimal to set the cash level $y_n(x)$ after a decision is made according to

62 $$\begin{aligned} y_n(x)=\left\{ \begin{array}{lll}T_n &{} \hbox {if} &{} x \le t_n \\ \in \{x,T_n\} &{} \hbox {if} &{} x \in (t_n,t'_n) \\ x &{} \hbox {if} &{} x \in [t'_n,u'_n) \\ \in [t'n,x] &{} \hbox {if} &{} x \in [u'_n,u_n) \\ U_n &{} \hbox {if} &{} x \ge u_n.\end{array}\right. \end{aligned}$$

In summary, Chen and Simchi-Levi (2009) followed a sequential decision-making approach using dynamic programming to minimize the total expected costs over the planning horizon. They proposed a model based on bounds, without assuming any particular density function for cash flows, but rather a general one. However, no practical application has been provided to illustrate the model using a real case.

### Baccarin (2009)

To the best of our knowledge, quadratic holdings and penalty costs have been considered for the first time in Baccarin (2002). Furthermore, a general multidimensional approach to the cash management problem was first introduced by Baccarin (2009) using generalized cost functions and providing theoretical results for two bank accounts. Baccarin considered cash management systems with multiple bank accounts, in which cash balances fluctuate as a homogeneous diffusion process in $\mathbb {R}^n$. They formulated the model as an impulse control problem with unbounded cost functions and linear costs.

The optimization problem considers an *n*-dimensional Wiener cash flow process $W_t$ that determines the dynamics of cash balances *x*(*t*) in the absence of any control action using the following Ito stochastic differential equation:

63 $$\begin{aligned} d x(t) = b (x(t)) dt + \sigma (x(t)) dW_t, \,\,\,\, x(0) = x \end{aligned}$$

where $b(x), \sigma (x) \in W^{1,\infty } (\mathbb {R}^n)$. Then, an impulse control strategy within a continuous time framework is a sequence of control actions $\xi _i$ at time $t_i$ to form policy $V=\{\xi _1,t_1; \ldots \xi _i,t_i; \ldots \}$ with $t_i \le t_{i+1}$. Subsequently, given policy *V*, the controlled process *y*(*t*) is defined as follows:

64 $$\begin{aligned} y(t) = y(0) + \int _0^t b(y(s))ds + \int _0^t \sigma (y(s)) dW_s + \xi _1 + \ldots + \xi _{\alpha _t} \end{aligned}$$

where $\alpha _t = {\text {max}}\{n|t_n \le t\}$. Holding costs are given by function *f*(*y*) and transaction costs by function $C(\xi )$, which is assumed to be lower semicontinuous and unbounded from above when $|\xi |\rightarrow \infty$. These holding and transaction cost functions satisfy the following inequalities:

65 $$\begin{aligned}{} & {} 0 \le f_0 < f(y) \le f_0(1+|y|^s), s>0 \end{aligned}$$

66 $$\begin{aligned}{} & {} 0 \le C < C(\xi ) \le d(1+|\xi |^p), p>0. \end{aligned}$$

As a result, each control policy *V* has an associated cost

67 $$\begin{aligned} J_x(V)=\texttt {E}\left\{ \sum _{i=1}^\infty C(\xi _i) \texttt {e}^{-\gamma t_i} \chi _{t_i < \infty } + \int _0^\infty \texttt {e}^{-\gamma s} f(y_x(s))ds \right\} \end{aligned}$$

where $\gamma >0$ is the discount rate and $\chi _{t_i < \infty } = 1$ if $t_i<\infty$, and zero otherwise. The problem is then to minimize $J_x(V)$ over the set *A* of admissible controls *V*. The optimal control is obtained by dividing $\mathbb {R}^n$ into two complementary regions: a continuation set, where the system evolves freely, and an intervention set, where the system is controlled in an optimal manner.

In summary, Baccarin (2009) provided a sound theoretical framework for cash management systems with multiple bank accounts within a continuous time framework with general cost functions and a single objective, namely, cost. Cash flows are assumed to follow a Wiener process, and the numerical solution to the optimization problem can be obtained by the finite element method, as described in Cortey-Dumont (1985), Boulbrachene (1998), which considers a discrete approximation of the continuous framework described above.

### Recent contributions: the operation’s research perspective

In this section, we refer to several recent cash management works (after 2000) that deserve a mention because of some interesting characteristics. In this sense, Hinderer and Waldmann (2001) formally introduced the concept of environmental uncertainty in CMP by providing a rigorous mathematical framework and exploring different cases of cash flow processes. Premachandra (2004) used a diffusion process as in Baccarin (2009) to propose a generalized version of the Miller and Orr (1966) model. Baccarin (2002) also considered quadratic holding costs for the first time in the cash management literature. Bensoussan et al. (2009) extended the model by Sethi and Thompson (1970) by applying a stochastic maximum principle to obtain the optimal cash management policy.

Melo and Bilich (2013) proposed an Expectancy Balance Model to minimize combined holding and shortage costs in an attempt to deal with uncertainty. This model considers the existence of both deterministic flows, which are known in advance, and stochastic flows, grouped into intervals of occurrence. Recently, da Costa Moraes and Nagano (2014) proposed the use of genetic algorithms, as in Gormley and Meade (2007) and particle swarm optimization to solve the CMP using the Miller and Orr (1966) model. They provide numerical examples using Gaussian cash flows for both solvers within the structure of a single bank account and two alternative investment accounts.

Salas-Molina et al. (2018) proposed a multi-objective approach to the CMP by considering not only the cost but also the risk of alternative policies using the Miller and Orr (1966) model and compromise programming (Zeleny 1982; Yu 1985; Ballestero and Romero 1998). They proposed the use of the standard deviation (and upper semi-deviation) of daily costs as a measure of risk. The third goal (stability) was proposed in Salas-Molina et al. (2020). In Salas-Molina et al. (2017), the authors showed that forecasting accuracy is highly correlated to cost savings in cash management when using forecasts and the Gormley and Meade (2007) model. The authors used different cash flow forecasters based on time-series features. A similar approach, based on machine learning was proposed by Moubariki et al. (2019) and Salas-Molina (2019), developed a machine-learning approach to fit cash management models to specific datasets.

Herrera-Cáceres and Ibeas (2016) proposed a model predictive control approach in which a given cash balance function is used as a reference trajectory to be followed by means of the appropriate control actions. In this proposal, cash managers aim to minimize deviations from a reference trajectory instead of minimizing any cost function. In contrast, Schroeder and Kacem (2019), Schroeder and Kacem (2020) described online algorithms to deal with interrelated demands for cash flows without making any assumptions about the probability distribution of cash flows. Finally, a formal approach to managing cash with multiple accounts based on the graph theory was proposed by Salas-Molina et al. (2021).

## A multidimensional analysis of the cash management problem

In the following section, we summarize the main cash management models presented in the literature according to the six dimensions introduced in Section 3, as shown in Tables 1 and 2.

1. **Models.** The use of Bound-Based Models (BBM), whose policies are determined by a set of level or bounds, is a common pattern. From the initial inventory approach to the CMP by Baumol (1952), most models have attempted to derive optimal policies within the framework of some simple policy, typically employing constant cash balance bounds. A slight departure of this framework was considered by Stone (1972) and Gormley and Meade (2007) to introduce forecasts as key inputs to a BBM model. A more practical approach was followed by Archer (1966) to focus on the statistical exploration of data to deal with the lack of synchronization of inflows and outflows. Recently, Baccarin (2009), Bensoussan et al. (2009) and Schroeder and Kacem (2020) proposed models without relying on bounds.
2. **Cash flow process.** A wide variety of cash flow processes have been considered in the literature, ranging from the uniform and perfectly known cash flow in Baumol (1952) and Tobin (1956), to purely stochastic behavior in Miller and Orr (1966), Eppen and Fama (1969), Constantinides and Richard (1978), Premachandra (2004), Baccarin (2009), da Costa Moraes and Nagano (2014), which usually implies a Gaussian distribution. The selection of any cash flow process implies the assumption of either a continuous time framework (Constantinides and Richard 1978; Baccarin 2009) or a discrete time framework (Stone 1972; Penttinen 1991; Gormley and Meade 2007). Data sets are hardly used with the exception of Salas-Molina et al. (2018).
3. **Cost functions.** The linear cost assumption is also a common pattern with the exception of Baccarin (2002, 2009), that considered more general cost functions. However, there also exist differences in the linear approach. While Baumol (1952) and Miller and Orr (1966) considered only fixed costs, Tobin (1956) and the subsequent works included fixed and variable costs in their models.
4. **Objectives.** It is also important to note that all models focus on a single objective, namely, cost, neglecting risk analysis. However, the works by Stone (1972), Hinderer and Waldmann (2001), Gormley and Meade (2007) are remarkable initial attempts to include uncertainty in the analysis of the best policies. The use of forecasts seems to be a sound strategy to reduce uncertainty in the CMP as shown in Salas-Molina et al. (2017). To handle the inherent uncertainty of cash flows, Salas-Molina et al. (2018) introduce the concept of risk analysis in a multi-objective approach to the CMP. Finally, Salas-Molina et al. (2020) extended the multi-objective approach to three different goals: cost, risk, and stability.
5. **Solvers.** There are also differences in the techniques used for solving the CMP. However, three solving techniques stand out: analytic solutions as in Baumol (1952), Tobin (1956), Miller and Orr (1966), Constantinides and Richard (1978), Hinderer and Waldmann (2001), Schroeder and Kacem (2020); dynamic programming as in Eppen and Fama (1969), Daellenbach (1971), Penttinen (1991), Chen and Simchi-Levi (2009); and approximate techniques as in Archer (1966), Stone (1972), Gormley and Meade (2007), da Costa Moraes and Nagano (2014).
6. **Bank accounts.** Although cash management systems with multiple bank accounts are the rule rather than the exception in practice, almost all previous models derive policies for a single bank account and provide no method to extend their results to multiple bank accounts. Only Baccarin (2009) approached the CMP from a multidimensional perspective to deal with multiple bank accounts. More recently, Salas-Molina et al. (2020) considered multiple bank accounts in the CMP and Salas-Molina et al. (2021) proposed a formal analysis of cash management models with multiple bank accounts based on graph theory.

**Table 1:** Characteristics of CMP models (I). F=fixed; V=Variable

Reference	Model	Cash flow	Cost function
Baumol (1952)	Deterministic	Uniform	Linear (F)
Tobin (1956)	Deterministic	Uniform	Linear (F,V)
Miller and Orr (1966)	Bound-based	Random walk	Linear (F)
Eppen and Fama (1969)	Bound-based	Random walk	Linear (F,V)
Daellenbach (1971)	Bound-based	Non-stationary	Linear (F,V)
Stone (1972)	Bound-based with forecasts	Predictable	None
Constantinides and Richard (1978)	Bound-based	Diffusion	Linear (F,V)
Penttinen (1991)	Bound-based	Exponential	Linear (F,V)
Gormley and Meade (2007)	Bound-based with forecasts	Empirical	Linear (F,V)
Chen and Simchi-Levi (2009)	Bound-based	Density function	Linear (F,V)
Baccarin (2009)	Unconstrained	Diffusion	Polynomial
Bensoussan et al. (2009)	Unconstrained	Known value	Linear (V)
da Costa Moraes and Nagano (2014)	Bound-based	Normal	Linear (F,V)
Salas-Molina et al. (2018)	Bound-based	Data set	Linear (F,V)
Schroeder and Kacem (2020)	Unconstrained	Bounded	Linear (F,V)
Salas-Molina et al. (2020)	Unconstrained	Predictable	Linear (F,V)

**Table 2:** Characteristics of CMP models (II)

Reference	Objective	Solver	Accounts
Baumol (1952)	Cost	Analytic	Single
Tobin (1956)	Cost	Analytic	Single
Miller and Orr (1966)	Cost	Analytic	Single
Eppen and Fama (1969)	Cost	Dynamic programming	Single
Daellenbach (1971)	Cost	Dynamic programming	Single
Stone (1972)	Cost	Simulation	Single
Constantinides and Richard (1978)	Cost	Analytic	Single
Penttinen (1991)	Cost	Dynamic programming	Single
Gormley and Meade (2007)	Cost	Evolutionary	Single
Chen and Simchi-Levi (2009)	Cost	Dynamic programming	Single
Baccarin (2009)	Cost	Finite element method	Multiple
Bensoussan et al. (2009)	Cost	Maximum principle	Single
da Costa Moraes and Nagano (2014)	Cost	Genetic algorithm	Single
Salas-Molina et al. (2018)	Cost and risk	Compromise programming	Single
Schroeder and Kacem (2020)	Cost regret	Min-max regret algorithm	Single
Salas-Molina et al. (2020)	Cost, risk, stability	Stochastic programming	Multiple

## Open research questions in cash management

From the previous review, it can be concluded that all relevant issues regarding cash management have been covered by the aforementioned cash management models. However, our taxonomy allows for the identification of open research questions in cash management, as we discuss next. From Table 1, we can infer that bound-based models seem to be a common pattern in cash management. However, recent proposals have questioned the use of bounds (Baccarin 2009; Herrera-Cáceres and Ibeas 2016) and probability distribution assumptions to derive optimal policies (Schroeder and Kacem 2019, 2020). Indeed, the ultimate goal of cash managers is not to find the best set of bounds but the best policy disregarding the required steps to achieve it. The utility of forecasts in cash management have been demonstrated in Gormley and Meade (2007) and Salas-Molina et al. (2017). These results must encourage cash managers to rely on time-series forecasting or machine learning techniques to reduce uncertainty in the near future.

In addition to its critical importance for real-world institutions, empirical cash flows are not common in cash management literature, with the exception of Emery (1981), Gormley and Meade (2007) and Salas-Molina et al. (2017), Salas-Molina et al. (2018). Common assumptions imply Gaussian, independent, and stationary cash flows in the form of a random walk or a diffusion process (Miller and Orr 1966; Constantinides and Richard 1978; Baccarin 2009). However, real-world cash flows may not accommodate such strong assumptions. As a result, the particular statistical properties of cash flows and their ability to predict them are research questions worth addressing.

The assumption of linear cost functions is not as restrictive in cash management as it may appear at first glance. However, considering piece-wise linear cost functions as in Katehakis et al. (2016) or even non-linear cost functions as in Baccarin (2002, 2009) may allow a better representation of real-world cash management problems. A closely related topic is the selection of risk measures when considering not only cost but also the risk of alternative policies as an additional objective in cash management, as suggested by Salas-Molina et al. (2018). The authors used the standard deviation of daily costs as a measure of risk; however, alternative measures of risk can also be explored (Artzner et al. 1999; Szegö 2002; Rockafellar and Uryasev 2002). Indeed, a comprehensive risk analysis of cash management represents an appealing research area in cash management.

Obtaining a policy that optimizes some objective functions is not straightforward. Beyond the discussion about the required assumptions to apply one technique or another, a rather unexplored issue is the optimality of the solutions provided by each method. While dynamic or linear programming ensure optimality, evolutionary algorithms, or particle swarm optimization they return only approximate solutions. However, there is a lack of supporting technology in the form of software applications for cash management that deserves the attention of the research community. The computing times, robustness of the solutions provided, and deployment costs of alternative methods are also worth exploring. From Table 2, we observe that only Baccarin (2009) and Salas-Molina et al. (2020) approached the cash management problem considering multiple bank accounts. Since the presence of several accounts is very common in practice, cash management models that can handle multiple bank accounts and transactions between them constitute an interesting topic.

It is important to highlight that open research questions do not arise by exploring only one dimension at a time. On the contrary, chances are that new research opportunities derive from a combination of values that received little attention of the research community. As an example, consider an unconstrained model using forecasts obtained from empirical cash flows that aim to minimize a multi-objective cost-risk function with piecewise linear cost functions through linear programming within a cash management system with multiple bank accounts.

The existence of multiple dimensions in CMP implies that the selection of cash management models, cost functions, solvers, and many other factors is a complex task. It seems clear that no cash management model is best for any decision-making context. As a result, the design of methodologies to select the appropriate models to solve CMP is an additional open research question. The set of all relevant operating conditions that are important in the decision-making context can be expressed as a set of parameters (Hernández-Orallo et al. 2013) that can ultimately be used to select models according to the preferences of practitioners. Multiple criteria decision-making techniques can help deal with multidimensional problems in finance. An example of the application of these techniques to the context of evaluating clustering algorithms in financial risk analysis can be found in Kou et al. (2014). More recently, Kou et al. (2021a) proposed the use of a hybrid multicriteria decision-making process in which different models were used to rank available alternatives.

Except for Salas-Molina et al. (2017) and Salas-Molina et al. (2018), the use of datasets and the application of forecasting models in cash management are scarce. We argue that time-series prediction models and other machine learning techniques may enhance decision-making in finance. We refer interested readers to recent reference books by Dixon et al. (2020) and Consoli et al. (2021), reviews by West and Bhattacharya (2016) and Henrique et al. (2019), and applications by Moubariki et al. (2019), Li et al. (2021), Kou et al. (2021b) and Manthoulis et al. (2021).

Finally, we must also point out that the integration of external factors, such as the impact of a financial crisis, in cash management models is also an interesting future line of research. In Section 2, we review the related literature on CMP from economic and financial perspectives. In Section 4, we review the most relevant contributions to CMP from the decision-making perspective. By combining these two approaches, we expect that cash management decision-making models can be enhanced with additional relevant factors. We consider this integration to remain an important open research question in cash management.

## Concluding remarks

In this study, we review the research literature relevant to the cash management problem since the first contribution by Baumol (1952) to the most recent contributions. We use this review to identify several research opportunities in cash management. We propose a new taxonomy based on the main dimensions of the cash management problem: (i)the model deployed, (ii)the type of cash flow process considered, (iii)the particular cost functions used, (iv)the objectives pursued by cash managers, (v)the method used to set the model and solve the problem, and (vi)the number of accounts considered. We use these six important dimensions as a framework to classify the most relevant contributions in cash management. Linking the dimensions with the reviews, we performed a multidimensional analysis of these contributions, which ultimately allowed us to highlight several open research questions in cash management. As a result, topics such as risk analysis in cash management, the utility of forecasts, and the possibility of handling multiple accounts have been identified as new research opportunities. Researchers may extend the number of dimensions, suggest new instances for each dimension, or even link unexplored instances to enrich the analysis of the cash management problem.

## Data Availability

not applicable.

## Abbreviations

CMP: Cash management problem
ATM: Automated teller machine
BBM: Bound based model
F: Fixed
V: Variable
